# Annotations

**Published:** 1892-08-13

**Authors:** 


### Annotations.

Almost every decently-educated Englishman on his return from a visit to France or Belgium has a struggle with his palate when he sits down to his own dinner table. The average English cook, as she is found in the houses of the middle class, is a babarian, and her mistress is in her hands like what John Baptist called contemptuously "a reed shaken with the wind." Cookery demands its Ruskin and its Matthew Arnold. If the average Englishman is totally ignorant of art, as Mr. Ruskin holds, and absolutely inaccessible to new ideas, as Mr. Matthew Arnold so incessantly affirmed, what, then, is the condition of the stupid, ignorant, and supremely self-satisfied cook of the middle-class household? She is, as Victor Hugo might have said, a dolt, a blockhead, a mass of the crassest obstinacy, a hopeless idiot. Let no reader suppose the writer to be in a bad temper. On the contrary, he is entirely calm, and is endeavouring to state with the most scientific precision exact facts about the English cook and her mistress; only he finds that the English tongue is not sufficiently strong in epithets and rich in phraseology to set forth the true state of the case. If any intelligent person wishes to know exactly how not to dine, he has but to look at the ordinary seven o'clock dinner of the middle-class Englishman. The soup, if there be any, may possibly be good, though it will pretty certainly be half cold. But the fish! The mass of it, the huge lump! The sauce for it—or, rather, the wall-paste! After the fish, the meat; the intolerable sirloin, or the maddening leg of mutton; cold, too, half the week! What philosophy can stand serene under such abominable monstrosities as these? Cooking is a lost art among us; or, rather, it is an art we never possessed, and do not now care to acquire. The mere cottager in France, the occasional chambermaid at a second-rate hotel, can beat our best middle-class cooks clean out of sight. It is pitiable! Will not Mr. James Payn, or Mr. Sala, or even the classical Mr. Andrew Lang come to the rescue? If the modern Englishman is threatened with early ruin in stomach, temper, and brains, it is because his cook is a hopeless fool, and his wife a helpless baby in her hands.

**How Not to Dine.**

It is said that Scotchmen claim all great men as their countrymen, and all great discoveries as their own. A Scottish policeman is said to have pointed out a monument erected to his countryman, as "James Watt, the Inventor of Steam." But they have been slow to do honour to a man who became a member of Watt's firm, and whom the same constable might have defined as "Alexander Murdoch, the Inventor of Gas." Perhaps it is because it was not in Scotland that Murdoch first made use of coal-gas for purposes of illumination, but in the village of Redruth, in Cornwall, that he has comparatively so little renown in his own country. But it was in his native county of Ayr that Murdoch—born just a hundred years ago, in 1792—made his first experiment. He was only a schoolboy when he was first struck with the manner in which the gas burst from the coal and ignited in his mother's fire. He took a small piece of live coal, put it in the bowl of a tobacco pipe, which he then covered up, and lit the gas at the end of the stem. Years passed before the boyish experiment led to any practical result, but when manager of the mines at Redruth he lighted the village with coal-gas. Public attention was first caught when, on the occasion of an illumination to celebrate the Peace of Amiens, the Soho Works, of which Murdoch was manager, were illuminated with gas. He met with some, but not overmuch, of the inventor's doom of misunderstanding and ingratitude. He was a successful man, and he has less to complain of than most, if while his discovery became every day more popular, his name was forgotten. Had Prometheus been allowed to take out a patent for the fire he stole from heaven, and died a Caucasian millionnaire, the tragic poets would have let him alone, and we should never have heard of him. Yet it is rather unfair that those who nightly profit by the results of Murdoch's genius should be ignorant to whom they owe the most convenient and cheapest of our illuminants—for so, in spite of all that the future may hold for electricity, it still remains. But with this centenary of his birth some renewed interest has been aroused in Murdoch's name, and his bust has been placed in the "Heroes' Room," in the Wallace Monument at Stirling. Perhaps he was not a hero, but it would be hard to deprive him of the title of a benefactor to his race.

**The Prometheus of Gas.**

The "City of Paris" has broken the record, hitherto held by the "Teutonic," in crossing the Atlantic. Mr. Tiffany has driven from Paris to Trouville, a distance of a hundred and thirty miles, in something under eleven hours, making the fastest drive on record. In our newspapers we constantly meet with paragraphs about the man who has made the best record in cycling, or running, or rowing, or billiard playing, or anything else under the sun. To make a record of one's own or beat that of somebody else is the ambition of a very large number of young men, and a rather serious ambition it is. The "City of Paris" has not long returned to its duties, having, whilst coming across the Atlantic at great speed, met with an accident, which it took many months to repair. Something similar might be told of a good many of our human record-breakers. Driving at the highest pressure possible, they meet with an accident which it takes a long time to repair, which in many cases is, indeed, not reparable. The mere breaking of bones is not alone, or even chiefly, to be feared. The strain on heart and lungs, though less obvious, is far more dangerous than any injury to bone and muscle. And to what end is all this striving? There are emergencies in which limb and life must be risked, but these have little to do with the making of records. To how many of the eight or nine hundred souls on board an Atlantic liner does it matter that they should reach Sandy Hook or Queenstown in twenty minutes less than was taken by the steamer that left last week? It is infinitely more important that they should be taken from one continent to the other without running unnecessary risks to gratify the vanity of the captain. And to whom does it matter that John Brown ran a mile in ten seconds less than John Smith? Why can they not use their strength and swiftness in something better than outstripping each other in turn until one or another breaks a blood-vessel, and so falls "out of the running." It is well known that a great amount of gambling goes on in steamers over the speed of the vessel; it is equally well known that our athletics are largely attended by betting. This is bad enough, and meets with sufficient reprobation; but it should be remembered that in both cases there is a gambling with life which is infinitely worse.

